# Duplication and expression patterns of *CYCLOIDEA*-like genes in Campanulaceae

**DOI:** 10.1186/s13227-021-00189-8

**Published:** 2022-02-06

**Authors:** Jingjing Tong, Eric B. Knox, Clifford W. Morden, Nico Cellinese, Fatima Mossolem, Aarij S. Zubair, Dianella G. Howarth

**Affiliations:** 1 Department of Biological Sciences, St. John’s University, Jamaica, NY USA; 2 Department of Biology, Indiana University, Bloomington, IN USA; 3 Department of Botany, University of Hawaiʻi at Mānoa, Honolulu, HI USA; 4 Florida Museum of Natural History, University of Florida, Gainesville, FL USA

**Keywords:** Campanulaceae, *CYCLOIDEA*, Flower symmetry, Gene expression, Gene duplication, Lobelioideae, Cyphioideae, Campanuloideae

## Abstract

**Background:**

*CYCLOIDEA (CYC)*-like transcription factors pattern floral symmetry in most angiosperms. In core eudicots, two duplications led to three clades of *CYC*-like genes: *CYC1*, *CYC2,* and *CYC3*, with orthologs of the *CYC2* clade restricting expression dorsally in bilaterally symmetrical flowers. Limited data from *CYC3* suggest that they also play a role in flower symmetry in some asterids. We examine the evolution of these genes in Campanulaceae, a group that contains broad transitions between radial and bilateral floral symmetry and 180° resupination (turning upside-down by twisting pedicle).

**Results:**

We identify here all three paralogous *CYC-like* clades across Campanulaceae. Similar to other core eudicots, we show that *CamCYC2* duplicated near the time of the divergence of the bilaterally symmetrical and resupinate Lobelioideae. However, in non-resupinate, bilaterally symmetrical Cyphioideae, *CamCYC2* appears to have been lost and *CamCYC3* duplicated, suggesting a novel genetic basis for bilateral symmetry in Cyphioideae. We additionally, utilized qRT-PCR to examine the correlation between *CYC*-like gene expression and shifts in flower morphology in four species of Lobelioideae. As expected, *CamCYC2* gene expression was dorsoventrally restricted in bilateral symmetrical flowers. However, because Lobelioideae have resupinate flowers, both *CamCYC2A* and *CamCYC2B* are highly expressed in the finally positioned ventral petal lobes, corresponding to the adaxial side of the flower relative to meristem orientation.

**Conclusions:**

Our sequences across Campanulaceae of all three of these paralogous groups suggests that radially symmetrical Campanuloideae duplicated *CYC1*, Lobelioideae duplicated *CYC2* and lost *CYC3* early in their divergence, and that Cyphioideae lost *CYC2* and duplicated *CYC3*. This suggests a dynamic pattern of duplication and loss of major floral patterning genes in this group and highlights the first case of a loss of *CYC2* in a bilaterally symmetrical group. We illustrate here that *CYC* expression is conserved along the dorsoventral axis of the flower even as it turns upside-down, suggesting that at least late *CYC* expression is not regulated by extrinsic factors such as gravity. We additionally show that while the pattern of dorsoventral expression of each paralog remains the same, *CamCYC2A* is more dominant in species with shorter relative finally positioned dorsal lobes, and *CamCYC2B* is more dominant in species with long dorsal lobes.

## Background

### Campanulaceae diversity

Campanulaceae, the bellflower family, are a large core eudicot group that encompasses roughly 2400 species in 84 genera [1]. They are found on six continents and many oceanic islands and are distributed from the tropics to the subarctic zones. There are at least three putative synapomorphic characters shared in Campanulaceae: laticifers, stamens attached to the disc of the ovary, and epigynous flowers [1]. The group is also recognized for its diversity in floral symmetry, resupination, and its pollination presentation mechanism.

The Campanulaceae are divided into five monophyletic subfamilies: Campanuloideae, Cyphioideae, Lobelioideae, Cyphocarpoideae, and Nemacladoideae [1]. Related groups to Campanulaceae are largely radially symmetrical, including the entirely radially symmetrical Rousseaceae. Campanuloideae have radially symmetrical flowers, while the other four clades have bilaterally symmetrical flowers [1, 2]. Campanuloideae includes approximately 1050 species in 50 genera. They are distributed worldwide, especially in temperate areas of the Old World, with the major centers of diversity in the Mediterranean Basin and the Middle East [1–3]. The Lobelioideae encompass about 1200 species in 29 genera [1]. They are also distributed nearly worldwide, with an origin in southern Africa [4], and a center of diversity in the New World tropics with a predominantly South American clade, “CBS (named for *Centropogon*, *Burmeistera*, and *Siphocampylus*),” containing roughly half the extant species [1, 5, 6]. Most species in Lobelioideae have resupinate (rotated 180° on the dorsoventral axis), bilaterally symmetrical flowers, connate (fused) stamens that form a staminal column tipped with an anther tube that releases pollen to the interior, and styles with brush hairs. The Lobelioideae exhibit a large diversification in growth-form, from small, herbaceous plants, to shrubs, to woody-rosette giant lobelias [7–10]. The Cyphioideae include 64 + species that are restricted to tropical and southern Africa. Cyphioideae and Campanuloideae are sister-groups [11], and share a simple pollen deposition mechanism [2]. The other three subfamilies (Lobelioideae, Cyphocarpoideae, and Nemacladoideae) weakly group together as a separate clade [2, 7, 11–13] The two smallest subfamilies, Cyphocarpoideae and Nemacladoideae, with smaller ranges and 3 and 25 species respectively [1], were not sampled in this study.

### Flower architecture and morphology

The symmetry of flowers is associated with their pollination, speciation, and diversification [14–16]. Floral symmetry can be classified into two main types: radially symmetrical (actinomorphic; polysymmetric), in which the flower has two or more central axes of symmetry; and bilaterally symmetrical (zygomorphic, monosymmetric), which have a flower with only one central axis of symmetry [14, 17]. Most asterid species have an additional complexity of partial corolla fusion, forming a sympetalous corolla tube proximally, and distinct petal lobes distally. Generally, bilaterally symmetrical flowers have floral organs of three different sizes or shapes (especially in the corolla lobes): dorsal (adaxial), lateral, and ventral (abaxial). In core eudicots, bilaterally symmetrical flowers most frequently have a corolla lobe arrangement of 2 dorsal lobes, 2 lateral lobes, and 1 ventral lobe (2 + 3 form). Other common forms include (4 + 1) and (0 + 5), with all of these types including a central ventral lobe pointed downward while the other four lobes shift in location [17].

**Fig. 1:**
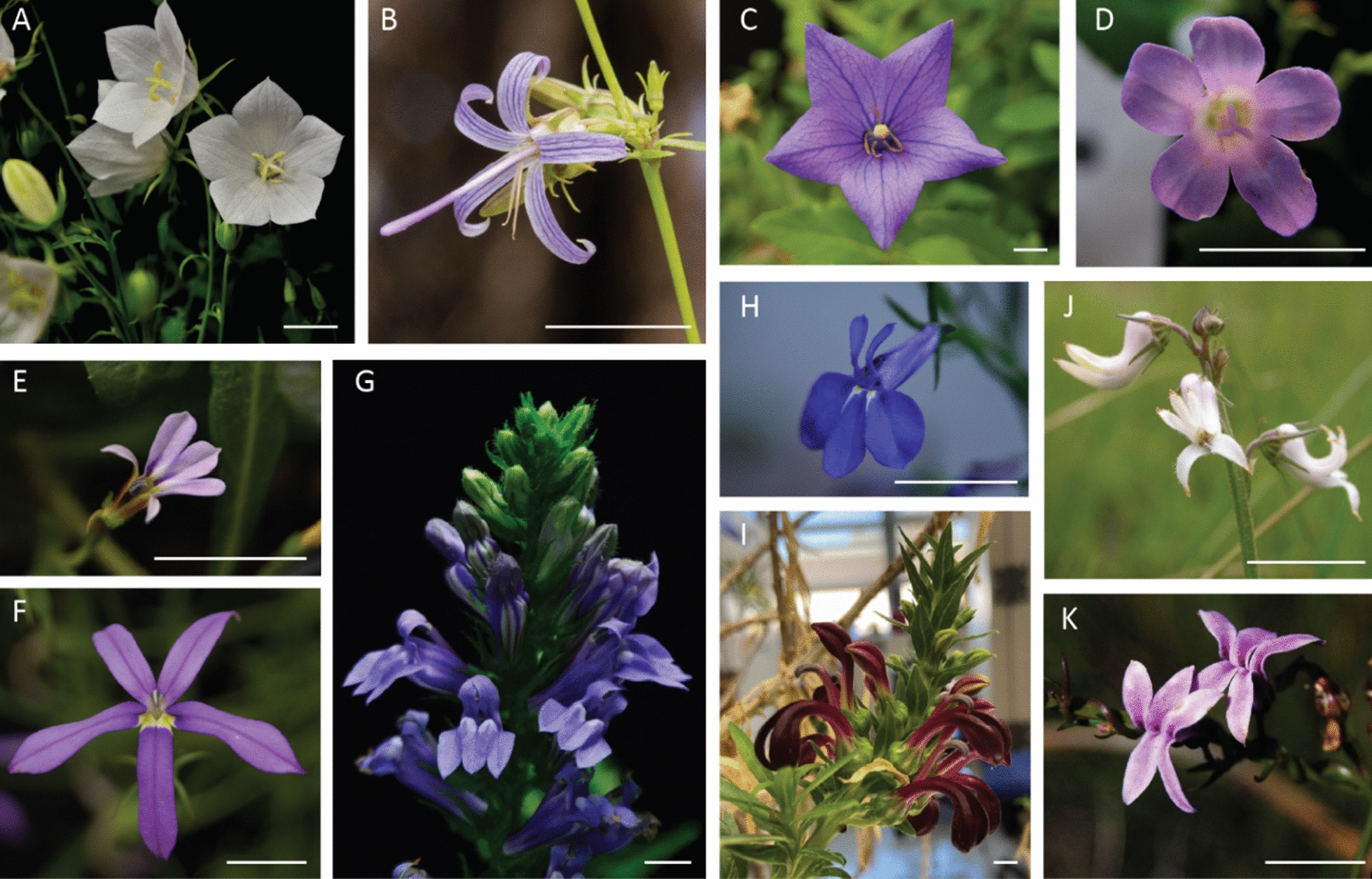
Campanulaceae species. **A**–**D** Campanuloideae species, have radially symmetrical flowers. **E**–**I** Nearly all Lobelioideae species, have different forms of resupinate, bilaterally symmetrical flowers. **J** and **K** Cyphioideae species, have bilaterally symmetrical flowers that are not resupinate. **A** *Campanula carpatica*, **B** Asyneuma prenanthoides*, **C** *Platycodon grandiflorus*, **D** *Campanula portenschlagiana*, **E** *Lobelia anceps*, **F** *Lithotoma axillaris*, **G** *Lobelia siphilitica*, **H** *Lobelia erinus*, **I** *Lobelia polyphylla*, **J** Cyphia longifolia**, **K** Cyphia longipetala**. *Photo right reserved to Marlin Harm. **Photo right reserved to Eric Knox. Other photos taken by Jingjing Tong

**Fig. 2:**
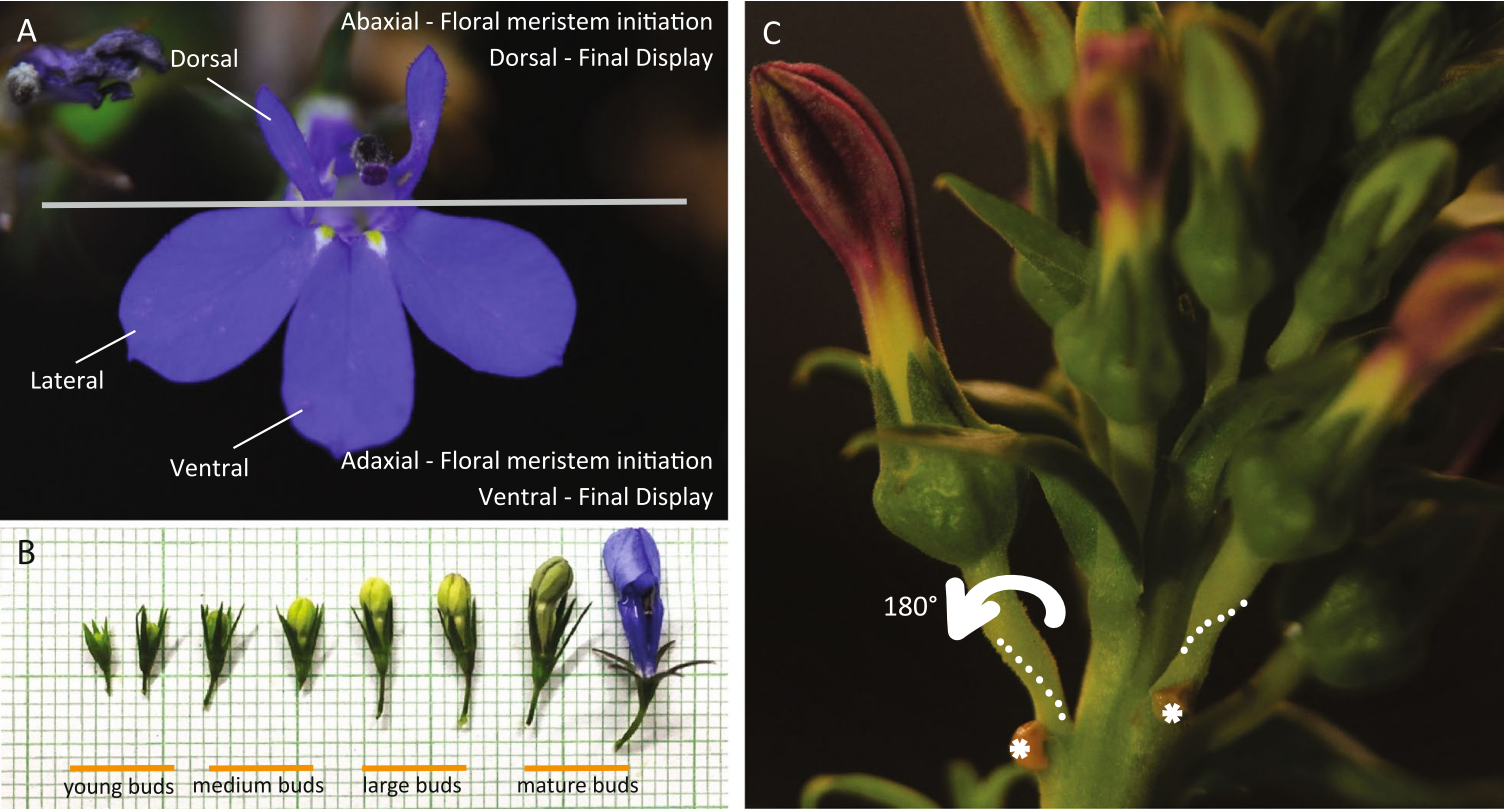
Lobelioideae flowers. **A** *Lobelia erinus* flower, **B** *L. erinus* floral buds in different stages. **C** *Lobelia polyphylla* large flower bud at the late development stage with removed bract. Asterisk marks location of latex exuding from removal of abaxial bract. Lobelioideae have resupinate flowers with the entire bud turning 180-degrees during development, resulting in the adaxial floral meristem region being the ventral region, and the abaxial floral meristem region being the dorsal region when the flower is mature. During floral bud growth, pedicels turn around at a relatively early stage of bud development and are completely turned 180-degrees upside-down by later stages of bud development. White dots show the location of the twist in the pedicels. Photos taken by Jingjing Tong

The ancestral flower symmetry of Campanulaceae remains equivocal given that Campanulaceae is divided into a bilaterally symmetrical clade (Lobelioideae, Cyphocarpoideae, and Nemacladoideae) and a clade with both bilaterally (Cyphoideae) and radially (Campanuloideae) symmetric lineages. Ancestral state reconstruction suggests the ancestor may have had bilaterally symmetrical flowers, with a reversal to radially symmetrical flowers in the Campanuloideae (Fig. 1A–D), [2], but related outgroups are nearly all radially symmetrical and the independent evolution of zygomorphy is equally plausible. In the other major clade, Lobelioideae, almost all species have resupinate flowers with (2 + 3) or (0 + 5) final floral displays (Figs. 1E–I, 2), except in *Monopsis* and two species of *Downingia*, in which flowers are not resupinate (with reversal to resupination in *M. decipiens*) [1, 18]. The remaining three subfamilies have non-resupinated flowers, with Cyphioideae (Fig. 1J, K) and Nemacladoideae having a (3 + 2) form, and Cyphocarpoideae having a (1 + 4) form [1, 2]. The shift in this family in both symmetry and resupination provide novel variation to examine how genes affect plant organ orientation and twisting of structures (Fig. 2).

Resupination in Lobelioideae occurs via the twisting of the pedicle after floral buds have formed [19]. Here we follow the terminology of Bukhari et al. [20] and use adaxial/abaxial to refer to the floral meristem orientation of the floral bud at developmental initiation, relative to the stem and subtending bract. We use dorsal/ventral to refer to the orientation of the final floral display (Fig. 2). Therefore, in resupinate Lobelioideae species, their abaxial region is in the dorsal position at anthesis.

### Genetic basis of floral symmetry

*CYCLOIDEA* (*CYC*) was the first characterized member of the floral symmetry gene regulatory network with a strong dorsal phenotypic effect, especially in the corolla and androecium [21–26]. In *Antirrhinum majus* (snapdragon), *CYC*-like genes are necessary to establish the dorsoventral axis in bilaterally symmetrical flowers [21]. *CYC* genes are members of the TCP gene family which exhibit high levels of sequence conservation in their TCP and R domains [27]. *CYC*, together with its paralog *DICHOTOMA* (*DICH*), co-express in the dorsal domain of the floral meristem from initiation and cause a reduction in growth of the corolla and stamens [28]. *Cyc-dich* double mutants in *A. majus* form radially symmetrical, ventralized flowers. Evidence from numerous comparative studies across flowering plants has shown that the duplication of *CYC*-like genes is highly correlative with the development of floral morphology in bilaterally symmetrical flowers [29–38]. Duplications or changes in the location or level of gene expression of *CYC*-like genes is highly associated with evolutionary shifts between radially symmetrical and bilaterally symmetrical flowers [39, 40].

Previous research has shown that two duplication events near the time of the diversification of the core eudicots produced three clades of *CYC*-like genes: *CYC1*, *CYC2* and *CYC3* [41]. Originally characterized *CYC* and *DICH* from *A. majus* are members of the *CYC2* clade and is widely involved in controlling bilateral symmetry across core eudicots [39, 41]. There are two or more paralogs of *CYC2* genes in nearly all characterized bilaterally symmetrical clades of core eudicots. In Asteraceae and Dipsacaceae, some clades have capitate inflorescences, which contain both radially and bilaterally symmetrical flowers. In these groups, there are multiple copies of *CYC2* genes, and they appear to be differentially expressed across the flowers of the inflorescence [29, 31–38, 40]. Additionally, the dorsoventral gradient of *CYC2* expression positively correlates with the level of bilateral symmetry [40]. In Asteraceae, the over-expression of *CYC2* genes can result in radially symmetrical disc flowers shifting to be more bilaterally symmetric [37].

The two additional paralogs of *CYC*-like genes have received less attention, although evidence indicates that they are also involved in inflorescence and/or floral patterning. The *CYC1* genes have not been shown to be directly involved in floral development, but *CYC1* genes may be responsible for plant or inflorescence branching architecture in *Arabidopsis*, *Populus*, and Asteraceae [37, 42–44]. The function of *CYC3* genes are still unclear in floral development [44]; however, based on gene expression data from Dipsacales and Asterales, *CYC3* genes may play a role in symmetry, at least in the campanulid (asterid II) clade [40, 45].

Given the resupinate flowers of the Lobelioideae, as well as the shifts in symmetry in the Campanulaceae sensu lato, we aimed to examine the evolutionary history of the Campanulaceae *CYC*-like (*CamCYC*-like) genes. We sampled *CamCYC* from Campanuloideae, Lobelioideae, and Cyphioideae species (Table 1) and compared them to molecular phylogenetic results for Campanulaceae [3, 7, 46]. The main aims in this study were to pinpoint the duplication events of *CamCYC*-like genes across Campanulaceae and use qRT-PCR to investigate the expression of *CamCYC2* genes in Lobelioideae species with resupinate, bilaterally symmetrical flowers. We examined expression in four species with different forms of bilateral symmetry and relative lobe lengths. *CamCYC2A* and *CamCYC2B* are highly expressed in the finally positioned ventral lobes (the adaxial side of the flower), suggesting conservation of dorsal identity in upside-down flowers. Additionally, individual copies of *CamCYC2* genes show different expression levels in different lobes, suggesting possible subfunctionalization between these copies.

**Table 1:** Campanulaceae species used in this study, including Genbank numbers for each sequenced gene/allele in each *CYC* gene clade

Clade	Label	Genus	Species	*CYC1*	*CYC2B*	*CYC2A*	*CYC3*
Campanuloideae	JC002(JT)	*Campanula*	*carpatica*	OM262907	OM263037		OM263196
Campanuloideae	JC008(JT)	*Campanula*	*cochleariifolia*	OM262908 OM262909			
Campanuloideae	3195(NC)	*Campanula*	*drabifolia*				OM263198
Campanuloideae	JC006(JT)	*Campanula*	*glomerata*	OM262910	OM263038		
Campanuloideae	CP011(JT)	*Platycodon*	*grandiflorus*	OM262918	OM263039		
Campanuloideae	CJ009(JT)	*Jasione*	*montana*	OM262915			
Campanuloideae	CJ001(JT)	*Campanula*	*persicifolia*	OM262911 OM262912			
Campanuloideae	JC007(JT)	*Campanula*	*portenschlagiana*	OM262913 OM262914			OM263197
Campanuloideae	CP012(JT)	*Phyteuma*	*scheuchzeri*	OM262916 OM262917	OM263036		
Cyphioideae	K4686	*Cyphia*	*comptonii*	OM262919			OM263203 OM263209
Cyphioideae	P5532	*Cyphia*	*digitata*	OM262920			OM263199 OM263200
Cyphioideae	K4725	*Cyphia*	*eckloniana*	OM262921			OM263207
Cyphioideae	K2340	*Cyphia*	*lasiandra*	OM262922			OM263210
Cyphioideae	K4734	*Cyphia*	*longipetala*	OM262924			OM263201 OM263204
Cyphioideae	P5461	*Cyphia*	*rogersii*	OM262925			OM263208
Cyphioideae	K4831	*Cyphia*	*smutsii*				OM263211
Cyphioideae	P5555	*Cyphia*	sp. nov	OM262923			OM263202 OM263205
Cyphioideae	K4726	*Cyphia*	*volubilis*	OM262926			OM263212
Cyphioideae	K4675	*Cyphia*	*zeyheriana*	OM262927			OM263206
Lobelioideae							
Genistoid E	K4216	*Lobelia*	*baumannii*	OM262958	OM263140	OM263055	
Genistoid E	K4942	*Lobelia*	*comptonii*	OM262965		OM263061	
Genistoid E	K4773	*Lobelia*	*dasyphylla*	OM262969		OM263063	
Genistoid E	K4314	*Lobelia*	*goetzei*	OM262973	OM263154	OM263066	
Genistoid E	K3316	*Lobelia*	*hartlaubii*	OM262976	OM263114	OM263067	
Genistoid E	K4814	*Lobelia*	*malowensis*	OM262987	OM263159	OM263070	
Genistoid E	K4609	*Lobelia*	*patula*	OM262991	OM263075	OM263076	
Genistoid E	P5475	*Lobelia*	*pteropoda*	OM262992		OM263079	
Genistoid E	K4634	*Lobelia*	*tomentosa*	OM263010	OM263177		
Genistoid E	K4654	*Lobelia*	*vanreenensis*	OM263014		OM263094	
Impares F	K5251	*Lobelia*	*cleistogamoides*	OM262962 OM262963			OM263215
Impares F	K5245A	*Lobelia*	*heterophylla*	OM262964			OM263213 OM263214
Impares F	K5210	*Colensoa*	*physaloides*	OM262935 OM262936			OM263218
Impares F	K5242	*Lobelia*	*rarifolia*	OM262994 OM262995		OM263080	
Impares F	K5259	*Lobelia*	*rhombifolia*	OM262996 OM262997		OM263081	
Impares F	K5253	*Lobelia*	*rhytidosperma*	OM262998		OM263084	
Impares F	K5279	*Lobelia*	*simplicicaulis*	OM263000 OM263002	OM263172	OM263087	
Impares F	K5234	*Lobelia*	*tenuior*	OM263007			OM263216
Impares F	A10185	*Lobelia*	*trigonocaulis*	OM263011		OM263091	OM263217
Impares F	K5252	*Lobelia*	*winifrediae*		OM263082	OM263083	
Monopsis G	K4628	*Monopsis*	*alba*	OM263023	OM263108	OM263107	
Monopsis G	K4790	*Monopsis*	*debilis*	OM263025			
Monopsis G	K4646	*Monopsis*	*decipiens*	OM263024		OM263104	
Monopsis G	K5116	*Monopsis*	*flava*	OM263026			
Monopsis G	K4402	*Monopsis*	*stellarioides*	OM263027	OM263109		
Monopsis G	P5246	*Monopsis*	*unidentata*	OM263028		OM263105 OM263106	
Broom H	P5621	*Lobelia*	*lasiantha*	OM262978			
Broom H	K4599	*Lobelia*	*linearis*	OM262980			
Grammatotheca I	K4642	*Lobelia*	*thermalis*			OM263089	
Erinoid L	K4951	*Lobelia*	*boivinii*			OM263057	
Erinoid L	K3475	*Lobelia*	*cymbalarioides*	OM262968		OM263062	
Erinoid L	JL002(JT)	*Lobelia*	*erinus*	OM262970	OM263152	OM263065	
Erinoid L	K4964	*Lobelia*	*inconspicua*			OM263068	
Erinoid L	K3401	*Lobelia*	*minutula*	OM262988		OM263073	
Erinoid L	K4841	*Lobelia*	*wilmsiana*	OM263017		OM263090	
Wimmerella M	K5276	*Lobelia*	*anceps*		OM263137		
Wimmerella M	K4685	*Wimmerella*	*bifida*	OM263035	OM263192		
Wimmerella M	K4594	*Wimmerella*	*hederacea*	OM263033	OM263193	OM263097	
Wimmerella M	K4545	*Wimmerella*	*pygmaea*	OM263034	OM263194	OM263101	
Wimmerella M	K5104	*Wimmerella*	*secunda*		OM263195	OM263102	
Mezlerioid3 N	K5182	*Lobelia*	*jasionoides*	OM262977	OM263155		
Mezlerioid3 N	K4566	*Lobelia*	*laurentioides*	OM262979	OM263157		
Mezlerioid3 N	K4589	*Lobelia*	*muscoides*	OM262989	OM263161		
Solenopsis O	Gr04/1	*Lobelia*	*urens*		OM263180	OM263092	
W North America P	K4663	*Downingia*	*bicornuta*	OM262948			
W North America P	UCBG770105	*Palmerella*	*debilis*	OM262946		OM263098	
W North America P	K4667	*Porterella*	*carnosula*	OM262947	OM263115	OM263099	
Diastatea Q	Wo8295	*Diastatea*	*micrantha*		OM263110 OM263111	OM263050	
E North America R	Jl007(JT)	*Lobelia*	*cardinalis*		OM263146	OM263060	
E North America R	K5282	*Lobelia*	*dortmanna*		OM263151		
E North America R	K2408	*Lobelia*	*fenestralis*	OM262949	OM263112		
E North America R	K5092	*Lobelia*	*puberula*		OM263113		
E North America R	JL003(JT)	*Lobelia*	*siphilitica*		OM263173	OM263088	
South America S	Ra s.n.2	*Burmeistera*	*crispiloba*	OM262930 OM262931		OM263042	
South America S	Lu15078	*Centropogon*	*comosus*	OM262932 OM262933	OM263119	OM263043	
South America S	JL005(JT)	*Lobelia*	*bridgesii*	OM262960 OM262961	OM263143	OM263058	
South America S	JL006(JT)	*Lobelia*	*polyphylla*		OM263164 OM263165	OM263078	
South America S	JL004(JT)	*Lobelia*	*tupa*	OM263012 OM263013	OM263178 OM263179		
Australasia T	RBGK2368	*Hypsela*	*Reniformis*	OM262942	OM263129		
Australasia T	A9820	*Isotoma*	*gulliveri*	OM262944 OM262945	OM263132		
Australasia T	K5237	*Isotoma*	*hypocrateriformis*	OM262943	OM263133	OM263134	
Australasia T	LI010(JT)	*Lithotoma*	*axillaris*		OM263130	OM263051	
Australasia T	W5440	*Lithotoma*	*petraea*	OM262954	OM263131	OM263052	
Australasia T	K5024A	*Lobelia*	*macrodon*	OM262985	OM263072	OM263071	
Australasia T	Ck2245	*Lobelia*	*pratioides*	OM262993	OM263166	OM263077	
Australasia T	K5027	*Lobelia*	*roughii*	OM262999	OM263167	OM263085	
Australasia T	K2369	*Pratia*	*arenaria*	OM262950 OM262951	OM263187		
Australasia T	W5265	*Pratia*	*gelida*	OM262952	OM263188		
Australasia T	A5357	*Pratia*	*pedunculata*	OM262953	OM263189	OM263100	
Giants U	NTBG970260	*Apetahia*	*longistigmata*	OM262928 OM262929	OM263116 OM263117		
Giants U	4561(CM)	*Brighamia*	*insignis*	OM262905 OM262906	OM263118	OM263040 OM263041	
Giants U	6799(CM)	*Clermontia*	*micrantha*		OM263120	OM263044	
Giants U	7011 (CM)	*Clermontia*	*persicifolia*	OM262934	OM263121	OM263045	
Giants U	1754(CM)	*Cyanea*	*acuminata*	OM262937 OM262938	OM263122	OM263046	
Giants U	K2375	*Cyanea*	*leptostegia*	OM262939 OM262940			
Giants U	2452(CM)	*Cyanea*	*superba*	OM262941	OM263123 OM263124		
Giants U	5416(CM)	*Delissea*	*rhytidosperma*	OM262896 OM262897 OM262903 OM262904	OM263125 OM263126	OM263047 OM263048	
Giants U	3835(CM)	*Delissea*	*subcordata*	OM262901 OM262902	OM263127 OM263128	OM263049	
Giants U	K706	*Lobelia*	*aberdarica*	OM262955 OM262956	OM263135 OM263136	OM263053	
Giants U	K731	*Lobelia*	*bambuseti*		OM263138 OM263139	OM263054	
Giants U	K220	*Lobelia*	*bequaertii*	OM262957 OM262959	OM263141 OM263142	OM263056	
Giants U	K802	*Lobelia*	*burttii*		OM263144 OM263145	OM263059	
Giants U	M2085	*Lobelia*	*columnaris*	OM262966 OM262967	OM263147 OM263148		
Giants U	K2353	*Lobelia*	*doniana*	OM262900	OM263149 OM263150	OM263064	
Giants U	K118	*Lobelia*	*giberroa*	OM262971 OM262972	OM263153		
Giants U	K698	*Lobelia*	*gregoriana*	OM262974 OM262975			
Giants U	K2381	*Lobelia*	*kauaensis*	OM262898 OM262899	OM263156		
Giants U	K3522	*Lobelia*	*longisepala*	OM262981		OM263069 OM263096	
Giants U	K623	*Lobelia*	*lukwangulensis*	OM262982 OM262983 OM262984	OM263158	OM263093	
Giants U	K426	*Lobelia*	*mildbraedii*		OM263160		
Giants U	K619	*Lobelia*	*morogoroensis*	OM262986		OM263074	
Giants U	NTBG910521	*Lobelia*	*niihauensis*	OM262990	OM263162		
Giants U	7162(CM)	*Lobelia*	*oahuensis*		OM263163		
Giants U	K610	*Lobelia*	*sancta*	OM263001	OM263168 OM263169	OM263086	
Giants U	RBGK5627	*Lobelia*	*sessilifolia*		OM263170 OM263171		
Giants U	K120	*Lobelia*	*stuhlmannii*	OM263003 OM263004	OM263174		
Giants U	K689	*Lobelia*	*telekii*	OM263005 OM263006			
Giants U	K876	*Lobelia*	*thuliniana*	OM263008 OM263009	OM263175 OM263176		
Giants U	4097(CM)	*Lobelia*	*villosa*	OM263015 OM263015 OM263022	OM263181 OM263182	OM263095	
Giants U	K262	*Lobelia*	*wollastonii*	OM263018 OM263019	OM263183 OM263184		
Giants U	K2379	*Lobelia*	*yuccoides*	OM263020 OM263021	OM263185 OM263186		
Giants U	4887(CM)	*Trematolobelia*	*kauaiensis*	OM263029 OM263031			
Giants U	4764(CM)	*Trematolobelia*	*macrostachys*	OM263030 OM263032	OM263190 OM263191	OM263103	

Included a total of 132 DNA samples, from 128 species, including 9 Cyphioideae species, 9 Campanuloideae species, and 110 Lobelioideae species

(JT = Jingjing Tong, NC = Nico Cellinese, CM = Clifford Morden, all other samples provided by Eric Knox) [75, 76]

## Results

### CamCYC1, CamCYC2, and CamCYC3 from Campanulaceae

**Fig. 3:**
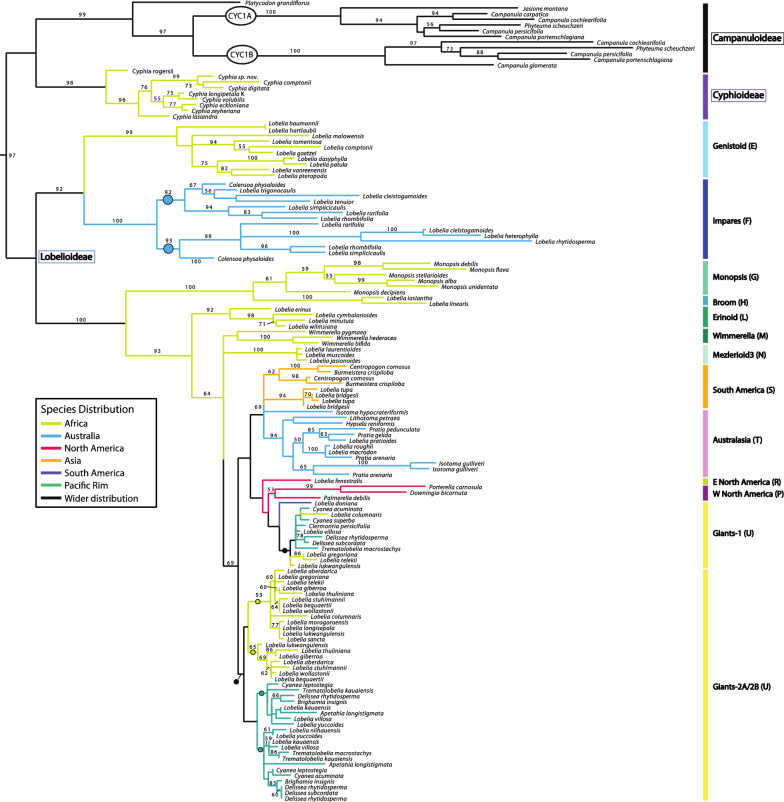
*CamCYC1* RAxML phylogenetic tree. Campanuloideae and Cyphioideae group into one clade, sister to Lobelioideae. The *CamCYC1* gene duplicated broadly within Campanuloideae, which is not shared with the other two subfamilies. Lobelioideae *CamCYC1* sequences are congruent with previously published Lobelioideae phylogenies [7, 10, 46, 47], with letter designations provided by Knox (unpublished). ML bootstrap values provided. Closed circles indicate hypothesized duplication

**Fig. 4:**
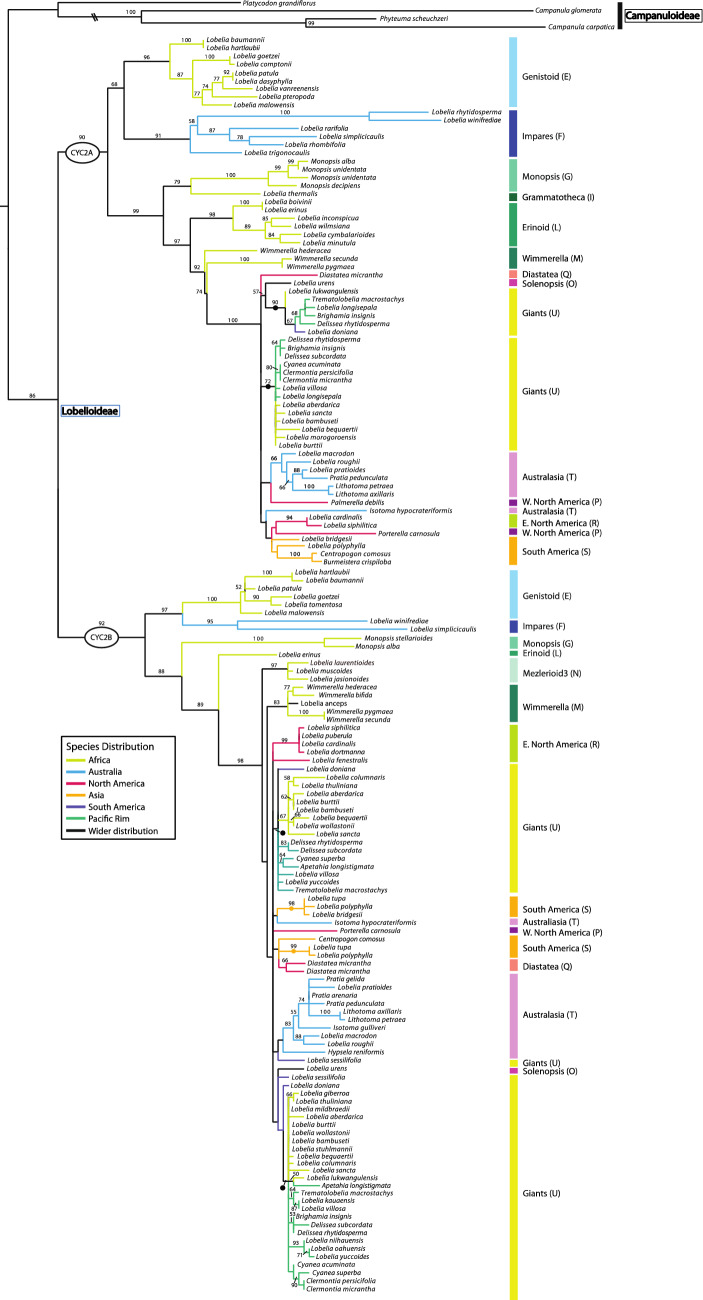
*CamCYC2* RAxML phylogenetic tree. The *CamCYC2* tree only includes sequences isolated from Lobelioideae and Campanuloideae, with no *CamCYC2* genes found in Cyphioideae. In Lobelioideae there is a clear duplication across the entire clade, which is not shared with Campanuloideae. The species relationship patterns are congruent between the two Lobelioideae subclades. The U clade includes multiple duplicate lineages in both *CamCYC2* paralogs. ML bootstrap values provided. Closed circles indicate hypothesized duplication

**Fig. 5:**
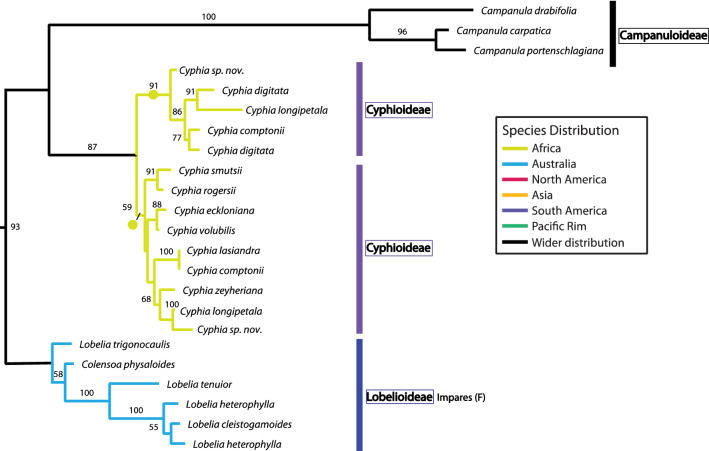
*CamCYC3* RAxML phylogenetic tree. ML bootstrap values provided. Closed circles indicate hypothesized duplication. Campanuloideae and Cyphioideae form a clade sister to Lobelioideae. A duplication is suggested in Cyphioideae. In Lobelioideae, only the F clade was recovered and is potentially lost from other lineages

Sequencing the TCP through R domains, we isolated *CamCYC1* from 83 Lobelioideae species, eight Campanuloideae species, and seven Cyphioideae species; *CamCYC2* from 90 Lobelioideae species and four Campanuloideae species; and *CamCYC3* from five Lobelioideae species, three Campanuloideae species, and eight Cyphioideae species. There were no *CamCYC2* gene sequences isolated from Cyphioideae. The tree topologies across the *CamCYC1*, *CamCYC2*, and *CamCYC3* clades were generally congruent with the estimated species phylogenies in these groups, especially in the best-sampled Lobelioideae. All three subfamilies were monophyletic and were consistent with a sister group relationship between Campanuloideae and Cyphioideae (Figs. 3, 4, and 5).

The *CamCYC1* sequence matrix was 492 bps long with 140 sequences, which included 12 sequences from Campanuloideae and nine sequences from Cyphioideae. Using midpoint rooting, Campanuloideae and Cyphioideae grouped in one clade, sister to the remaining 119 sequences, all isolated from 83 Lobelioideae species (Fig. 3). Our data support a duplication in *CamCYC1* within the Campanuloideae that is not shared with the other subfamilies (ML bootstrap = 100), with *Campanula cochlearifolia*, *C. persicifolia*, *C. portenschlagiana*, and *Phyteuma scheuchzeri* occurring in both clades. Cyphioideae *CamCYC*1 sequences form a single clade, although multiple *CamCYC1* gene sequences were isolated from most Cyphioideae samples, likely due to allelic diversity.

Lobelioideae *CamCYC1* formed a single clade with no broad duplication detected across Lobelioideae. Species distribution in Lobelioideae *CamCYC*1 subclades are congruent with previously published Lobelioideae phylogenetic relationships [7, 10, 46, 47]. Clade names and letter designations used are from [7, 10, 46, 47] and Knox (unpublished data), with Genistoid (E) and Impares (F) subclades forming a clade sister to the remaining samples. The Impares (F) subclade appears to have duplicated *CamCYC1* (ML bootstrap = 92) with 5 out of 10 sampled species (*Lobelia cleistogamoides*, *L. simplicicaulis*, *L. rhombifolia*, *L. rarifolia*, and *Colensoa physaloides*) yielding two highly differentiated copies. The U subclade, often called the giant lobelias, in some cases yield three highly differentiated sequence copies. The duplicated copies in the U2-A and U2-B subclades are possibly a result of this group being ancient tetraploids [48, 49]. There is no obvious explanation for the copies that comprise the U1 subclade, which is weakly embedded in a clade with members of the P, R, S, and T subclades.

The *CamCYC2* matrix was 363 bps long with 160 sequences, which included 156 sequences from Lobelioideae and only four sequences isolated from Campanuloideae (Fig. 4). No *CamCYC2* sequences were obtained from any Cyphioideae species despite targeted amplification. In the *CamCYC2* gene tree (Fig. 4), sequences from Lobelioideae species formed two clades, both of which were broadly congruent with the hypothesized species relationships: *CamCYC2A* (ML bootstrap = 92) and *CamCYC2B* (ML bootstrap = 90). Species relationships in both Lobelioideae *CamCYC2* clades shared a similar pattern and also corresponded with the *CamCYC1* tree. In the *CamCYC2* tree, sequences from 43 Lobelioideae species were found in both *CamCYC2A* and *CamCYC2B* clades. There were separate duplications into two U subclades in each of the *CamCYC2* paralogs. Our data indicate that *CamCYC2* genes have duplicated in the Lobelioideae, a duplication that does not appear to be shared with the *CamCYC2*-like genes isolated from Campanuloideae.

The *CamCYC3* gene tree included species from all three sampled subfamilies (Fig. 5). The *CamCYC3* matrix was 329 bps long with 23 sequences, including three sequences from Campanuloideae, 14 sequences from Cyphioideae, and six sequences from Lobelioideae. Fewer *CamCYC3* sequences were recovered compared to the other *CamCYC* genes. The three Campanuloideae sequences formed a clade. Sequences from Cyphioideae grouped into two clades, and sequences from three species (*Cyphia longipetala*, *C. sp. nov.,* and *C. comptonii*) in both clades suggests that *CamCYC3* duplicated sometime during the evolution of *Cyphia*. The very limited recovery of *CamCYC3* sequences from the Lobelioideae suggests that this gene has been lost in the subfamily except for the Impares (F) clade.

### Expression of *CamCYC2* genes in Lobelioideae species

**Fig. 6:**
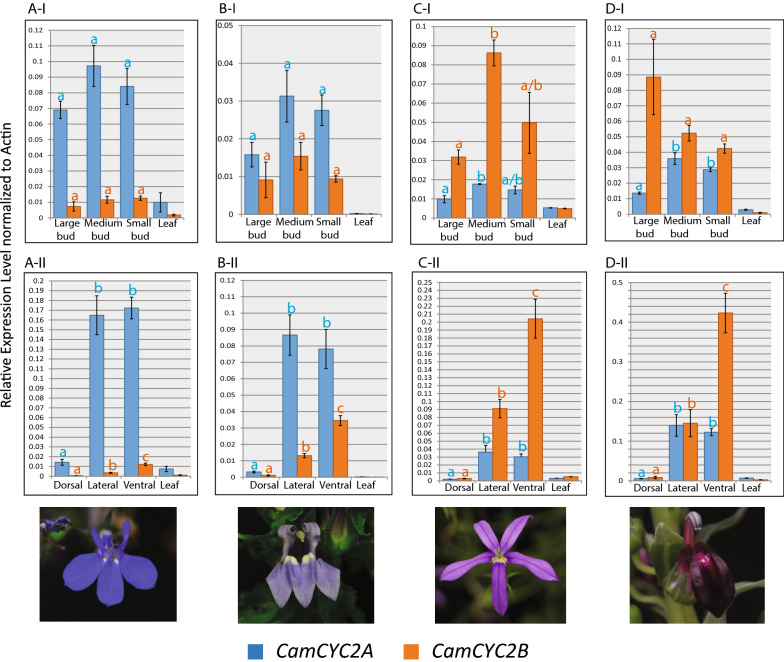
Relative expression levels of *CamCYC2A* (blue) and *CamCYC2B* (orange) genes in Lobelioideae species. **A** *Lobelia erinus*, **B** *Lobelia siphilitica*, **C** *Lithotoma axillaris*, **D** *Lobelia polyphylla*. **A-I**, **B-I**, **C-I**, **D**-**I** show *CamCYC2* genes in different floral bud stages, both *CamCYC2* genes are expressed through the whole floral growth stage; **A-II**, **B-II**, **C-II**, **D-II** show *CamCYC2* genes in different corolla lobes from medium buds. *CamCYC2A* is highly expressed in the ventral and lateral lobes, exhibiting lower expression in dorsal lobes; *CamCYC2B* is highly expressed in the ventral lobe, exhibiting intermediate expression in lateral lobes and low expression in dorsal lobes. Lines of the Y-axis are labeled with the same scale across all diagrams except (**D-II**). Y-axis is the relative expression level, normalized to *CamACTIN* as the reference gene. Levels of expression of a single paralog with statistically significant differences (p ≤ 0.05) across different tissues are indicated by separate letters, a, b, or c. All species resupinate at maturity

*CamCYC2A* and *CamCYC2B* expression levels were assayed with qRT-PCR across four Lobelioideae species with different floral morphologies, *Lobelia erinus* (Africa), *Lo. siphilitica* (North America), *Lithotoma axillaris* (Australia)*,* and *Lo. polyphylla* (South America). All four species have typical resupinate Lobeliaceae flowers with a final display having a medial ventral petal lobe (Fig. 6), and all expression patterns are described using dorsal and ventral position of that final display. The overall expression patterns were broadly similar across all four species (Fig. 6A-I, B-I, C-I, D-I), although, the expression levels between the two paralogs varied. In all species, *CamCYC2A* and *CamCYC2B* are strongly expressed in flowers and not leaves, and in most cases the expression levels were not statistically significant across flower bud stages. In the dorsal, lateral, and ventral corolla-lobe dissections across all four species, both *CamCYC2A and CamCYC2B* were highly expressed in the ventral region (adaxial initiation), and only minimally expressed in the dorsal region (abaxial initiation). *CamCYC2A* was expressed similarly in lateral and ventral lobes and significantly reduced in dorsal lobes. *CamCYC2B* was expressed in a gradient, with the highest expression in the ventral lobe, medium expression in lateral lobes, and lowest expression in dorsal lobes. This pattern was consistent among all four species, but which paralog predominated varied among species (Fig. 6A-II, B-II, C-II, D-II).

*Lobelia erinus* has resupinate flowers with the smallest size ratio of dorsal to lateral and ventral corolla lobes, with the lateral and ventral lobes similar in size, and the dorsal lobes about 15–20% as large (Figs. 2A, 6). *CamCYC2* genes are highly expressed in the lateral and ventral lobes (adaxial initiation) and have extremely low expression in the dorsal lobes (abaxial initiation; Fig. 6A-II). *CamCYC2A* is highly expressed in lateral and ventral lobes at similar levels (*p* = 0.762). By contrast, the dorsal lobe expression is significantly lower (dorsal/lateral *p* = 0.0017; dorsal/ventral *p* = 0.0002). *CamCYC2B* shows a dorsoventral gradient of expression, being most highly expressed in the ventral lobe, moderately expressed in the lateral lobes, and only minimally expressed in the dorsal lobes. The expression of *CamCYC2B* was significantly different in the three corolla lobe types (dorsal/lateral p = 0.006; dorsal/ventral p = 0.0008; and lateral/ventral *p* = 0.0031). Dorsal lobe expression in both *CamCYC2A* and *CamCYC2B* was similar to leaf expression. Temporally, *CamCYC2A* and *CamCYC2B* genes express in very early stages of flower development, and steadily express through bud growth stages, with no significant differences in expression levels in either gene (Fig. 6A-I). Comparing the two paralogs, *CamCYC2A* is much more highly expressed than *CamCYC2B* in floral tissue and flower buds in *Lo. erinus*. For instance, *CamCYC2A* expression is roughly 15 times higher than that of *CamCYC2B* in the ventral lobe (Fig. 6A-II).

*Lobelia siphilitica* has resupinate flowers with relatively large dorsal lobes compared with *Lo. erinus*, and dorsal lobes are about 40% the size of lateral and ventral lobes (Figs. 1G, 6). The expression patterns of *CamCYC2A* and *CamCYC2B* are similar to that of *Lo. erinus*. In *Lo. siphilitica*, *CamCYC2A* is highly expressed in a similar level in lateral and ventral lobes (*p* = 0.6438), and barely expressed in dorsal lobes (dorsal/lateral p = 0.0025 and dorsal/ventral *p* = 0.0032 (Fig. 6B-II). *CamCYC2B* is expressed most highly in the ventral lobe, intermediately in the lateral lobes, and extremely minimally in the dorsal lobes (dorsal/lateral *p* = 0.0009, dorsal/ventral *p* = 0.0004, and lateral/ventral *p* = 0.0029) (Fig. 6B-II). Temporally, *CamCYC2A* and *CamCYC2B* genes express in very early stages of flower development, and steadily express through bud growth stages, with no significant differences in expression levels in either gene (Fig. 6B-I). Similar to in *Lo. erinus*, *CamCYC2A* is more highly expressed than *CamCYC2B* in floral tissue and flower buds in *Lo. siphilitica*, but, with less of a differential between the paralogs. For instance, *CamCYC2A* expression is only roughly two times greater in the ventral lobe than that of *CamCYC2B* (Fig. 6B-II).

All five corolla lobes of resupinate *Lithotoma axillaris* flowers have similar size and shape, but the orientation of the staminal column (with filaments adnate to the fully connate corolla tube, which lacks the dorsal slit to the base typical of *Lobelia*) makes these flowers bilaterally symmetrical (Figs. 1F, 6). *CamCYC2A* and *CamCYC2B* have much higher expression in lateral and ventral lobes and are barely expressed in dorsal lobes, with overall expression patterns similar to *Lo. erinus* and *Lo. siphilitica* (Fig. 6C-II). *CamCYC2A* is significantly less expressed in dorsal lobes than lateral (*p* = 0.0163) and ventral (*p* = 0.0015) lobes, with similar expression between dorsal and lateral lobes (*p* = 0.5634). In *CamCYC2B* each lobe type has significantly different expression along a gradient as in the other species, with ventral/lateral (*p* = 0.0139), ventral/dorsal (*p* = 0.0012), and lateral/dorsal (*p* = 0.0016). Temporally, both paralogs are expressed early and continued to be expressed through development but with a statistically significant decrease in expression in large buds (Fig. 6C-I). In *CamCYC2A* the difference in expression in large bud versus medium bud is statistically significant (*p* = 0.0136), while other comparisons are not (large bud/small bud *p* = 0.1508 and medium bud/small bud *p* = 0.2016). *CamCYC2A* is consistently expressed through the bud development, with only a slight up-regulation in the medium buds. *CamCYC2B* expression peaks in the medium buds, and then decreases in large buds (*p* = 0.0021). A major difference in expression between *Li. axillaris* and previously discussed *Lobelia* species is that *CamCYC2B* is more highly expressed overall, compared to *CamCYC2A* in floral buds and corolla lobes (more than 7 times greater in ventral lobes). Therefore, the overall expression patterns among lobe types are the same across species, but the gene copy that is the most highly expressed flips.

*Lobelia polyphylla* has resupinate flowers with dorsal, lateral, and ventral lobes that are similar in size and shape, with the dorsal lobes slightly longer than the lateral and ventral lobes (Figs. 1I; 6). Additionally, all five lobes bend downward and away from the staminal column (toward the finally positioned ventral region in these resupinate flowers). *CamCYC2A* and *CamCYC2B* have significantly higher expression in lateral and ventral lobes and are barely expressed in dorsal lobes, with expression patterns similar to *Lithotoma axillaris* (Fig. 6D-II). *CamCYC2A* is expressed significantly less in dorsal lobes than lateral (*p* = 0.0079) and ventral (*p* = 0.0002) lobes, with similar expression between dorsal and lateral lobes (*p* = 0.5783). In *CamCYC2B* there is a dorsoventral gradient of expression, highest in ventral corolla lobes, with each lobe type having significantly different expression between dorsal/lateral (*p* = 0.0161), dorsal/ventral (*p* = 0.0011), and lateral/ventral (*p* = 0.0099) lobes. Temporally, both paralogs are expressed early and continue to be expressed through development, but with a statistically significant decrease in expression in large buds in only *CamCYC2A* (Fig. 6D-I). In *CamCYC2A*, expression in large buds is significantly less than that of small buds (*p* = 0.0004) or medium buds (*p* = 0.0042). In *Lo. polyphylla CamCYC2B* is more highly expressed than *CamCYC2A*, similar to the pattern observed in *Li. axillaris*, with *CamCYC2B* roughly 3.5 times more highly expressed than *CamCYC2A* in the ventral lobe.

## Discussion

**Fig. 7:**
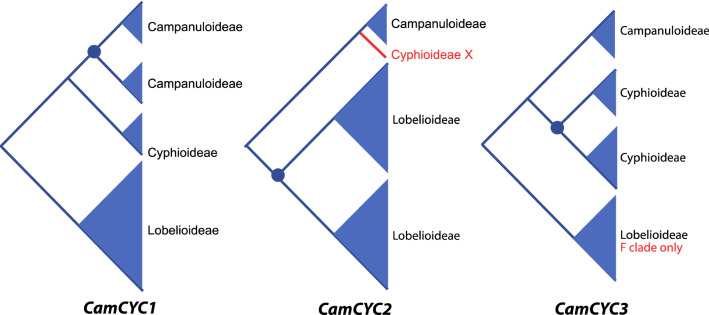
Summary *CYC*-like duplication events across Campanulaceae. *CamCYC1* duplicated in Campanuloideae and might have narrower duplications in the F and U clades in Lobelioideae. *CamCYC2* showed a clear duplication event specific to Lobelioideae and an apparent loss in Cyphioideae. *CamCYC3* duplicated in Cyphioideae and is apparently lost in all but one clade of Lobelioideae. *CamCYC3* might play a key role in bilateral symmetry instead of *CYC2*-like genes in Cyphioideae. Blue dots indicate hypothesized location of broadly duplicated clades

The three subfamilies of Campanulaceae sampled in this study have distinctly different floral symmetry modifications with radially symmetrical flowers in Campanuloideae, non-resupinate bilaterally symmetric flowers in Cyphioideae, and bilaterally symmetric flowers that are predominately 180° resupinate in Lobelioideae [1, 2, 7]. In these three groups, we uncovered broad gene duplications and losses that correlate with these morphological shifts. We detected all three core eudicot *CYC-*like genes from the *CYC1*, *CYC2*, and *CYC3* clades [41]. *CamCYC1* was thoroughly sampled from all three subfamilies, while *CamCYC2* was likely lost in Cyphioideae and *CamCYC3* was likely lost from all except the Impares (F) subclade of Lobelioideae (which along with the Genistoid (E) subclade is sister to the rest of the subfamily; Knox 2014). Additionally, we found evidence for subfamily duplications—*CamCYC1* duplicated in Campanuloideae, *CamCYC2* duplicated in Lobelioideae, and *CamCYC3* duplicated in Cyphioideae (Fig. 7).

### *CamCYC1* duplicated in the radially symmetric Campanuloideae

*CamCYC2*—the Campanulaceae member of *CYC2*, which is a clade that has shown functional conservation in patterning floral bilaterally symmetry [28, 30, 41, 50, 51]—was present across the radially symmetrical Campanuloideae, from four species that span the major clades of the group (Fig. 3). There was no evidence for duplications in *CamCYC2*, which is consistent with other radially symmetrical groups [39, 41]. Additionally, Campanuloideae *CamCYC2* copies had high sequence diversity, being on very long branches, and were therefore difficult to align with Lobelioideae species (Fig. 4). In other lineages with both radially symmetrical and bilaterally symmetrical flowers, such as Fabales, Malpighiales, and Dipsacales, species with radially symmetrical flowers have *CYC2*-like genes expressed uniformly across the whole corolla or have lost floral expression entirely [36, 38, 40, 52–58].

*CamCYC*3 was also found in Campanuloideae, but only in the C2 clade [3] and also on a long branch compared to Cyphioideae and Lobelioideae sequences (Fig. 5). *CYC3* has been shown to be involved in axillary bud outgrowth [44] and in floral symmetry [40], but with variable function in different plant groups.

The Campanuloideae show the most diversification in *CamCYC1* genes, with a duplication possibly shared across the Campanuloideae clade (Figs. 3, 7). With only minor differences, these duplicate gene trees agree with the estimated Campanuloideae species phylogeny [2, 3]. Studies in plant groups across core eudicots suggest that *CYC1* genes are functionally conserved, regulating the number and position of axillary bud development [42–44], as well as inflorescence architecture and development [37]. Loss-of-function mutants in *Arabidopsis* and *Populus* lead to a marked increase in bud outgrowth and plant branching [42–44]. It is possible that the duplication of *CamCYC1* set the stage for the variation in plant and inflorescence architecture in Campanuloideae. Flowers vary from solitary to complex inflorescences such as capitulate heads [1]. Broad duplications in *CYC1* are less common than in the other *CYC* clades, although they are consistently duplicated in lineages known for capitulate heads such as in the Asteraceae [31, 45], Dipsacaceae [59], and *Actinodium* [35].

### Cyphioideae have lost *CamCYC2* and duplicated *CamCYC3*

Cyphioideae typically have non-resupinate bilaterally symmetrical flowers with a 3 + 2 form, with one dorsal lobe, two lateral lobes, and two ventral lobes. In all other core eudicot bilateral symmetrical lineages studied to date, *CYC2* is differentially expressed across the dorsoventral axis and functions to pattern that bilateral symmetry [39]. Occasionally, *CYC2* genes appear to lose floral expression or be lost from the genome of certain species, however, these are always marked by shifts to radial symmetry [53, 54, 56, 57]. Additionally, in almost all cases, *CYC2* genes are duplicated in bilaterally symmetrical lineages [39, 41]. Here we report the first case of an apparent loss of *CYC2* in a bilaterally symmetrical core eudicot group, Cyphioideae (Fig. 7). Sampling nine species with multiple primer sets, no *CamCYC2* sequences were found, despite easily recovering them from Campanuloideae and Lobelioideae.

Along with a likely loss of *CYC2* in Cyphioideae, *CamCYC3* appears to be duplicated in this lineage (Figs. 5, 7). This is in stark contrast to Lobelioideae, which appear to have lost *CamCYC3* in all but the Impares (F) clade, with no evidence of gene duplication. *CYC3* is the most understudied paralog across core eudicots and also appears to be the most variable in function. *CYC3* genes are duplicated in some groups such as Dipsacales and Asteraceae [45, 59, 60], but have been likely lost in others such as Leguminosae and Gesneriaceae [61, 62]. In *Arabidopsis* (Brassicaceae) and *Populus* (Salicaceae), *CYC1* (*Branched1*) and *CYC3* (*Branched2*) orthologs have redundant function in regulating bud outgrowth [44, 63] with an increase in branching in loss-of-function mutants. Interestingly, *branched1* had the stronger phenotype in *Arabidopsis* [44] and *branched2* had the stronger phenotype in *Populus* [63]. Although *CYC3* gene function in floral symmetry has not previously been shown, studies in Dipsacales and Asteraceae reported expression patterns that are suggestive of this role, with dorsoventral expression of *KmCYC3B* in *Knautia macedonica* [40] and *HaCYC3a* expression specific to ray florets in *Helianthus annuus* [37]. This evidence suggests that *CYC3* function is highly labile. Additionally, function specific to plant branching appears to be found in rosids while floral expression has been seen in campanulid asterids. *CYC3* could play a role in floral symmetry in campanulids such as *Cyphia*, and is possibly filling the role of the lost *CYC2*.

Even though Cyphioideae are bilaterally symmetrical, their genetic signature compared to other core eudicot species would actually suggest they are radially symmetrical, with an apparent loss of functional *CYC2-like* genes. In the latest phylogenetic analyses [2] Cyphioideae are sister to radially symmetrical Campanuloideae, which retain *CYC2*-like genes, however, they are highly diverged. Additionally, Cyphioideae are not resupinate as are most species of Lobelioideae. However, unlike the lobe arrangement of most bilaterally symmetrical core eudicots, *Cyphia* flowers (Fig. 1J, K) have 3 dorsal corolla lobes and 2 ventral lobes. Standard orientation of core eudicot bilaterally symmetrical flowers have a single ventral corolla lobe, pointed downward, with two lateral and two dorsal lobes each acting as pairs that can shift along the dorsoventral axis in tandem [17]. Campanuloideae and Lobelioideae have medial ventral petal lobes [64], but the later only after resupination via torsion of the pedicel [65, 66]. The lobe arrangement in Cyphioideae, with a 3 + 2 corolla lobe arrangement, necessitates a shift in that axis at some point early in development, possibly through an independent resupination event or differentiation in the location of primordial initiation. The latter is suggested by Leins and Erbar [67] with initiation of petal lobe primordia in a 3 + 2 arrangement, however, with asymmetric early development across the dorsoventral axis. Therefore, these data support the hypothesis that the ancestral Campanulaceae was radially symmetrical and that the genetic programming of bilateral symmetry likely evolved independently in Cyphioideae and Lobelioideae.

### In Lobelioideae, *CamCYC1* duplicated within two subclades while *CamCYC3* appears to be lost in all but the Impares clade

In Lobelioideae, *CamCYC1* is broadly congruent with the hypothesized species phylogeny with no obvious subfamily-wide duplications (Figs. 3, 7) [7–10, 46, 47]. There are multiple sequences in a few species; however, these are likely alleles or more recent isolated duplications. *CamCYC1* has not been implicated in bilateral symmetry in any groups, instead being involved in plant and inflorescence branching in several lineages [44, 63, 68]. *CamCYC1*, in keeping with the general paucity of CYC1 gene duplications found in other groups, lacks the broad duplication pattern commonly seen in *CYC2* and *CYC3* genes correlating with a shift to bilateral symmetry [45, 60, 62]. However, there are duplications found in the Impares (F) clade as well as the giant lobelioids (U), likely due to independent ancient genome duplications [48, 49, 69].

The Impares clade, appearing to have duplicated *CamCYC1* early in its diversification (Fig. 3), is notable for having a diversity of chromosome numbers, varying among 8, 9, 10, and 11 [69], while most of Lobeliaceae have multiples of 7 chromosomes. This suggests that a genome duplication occurred early in the diversification of the Impares clade, followed by subsequent frequent chromosome losses. The duplication in *CYC1* likely correlates with that genome duplication; however, we have no hypothesis for why these genes were maintained in this lineage. The Impares clade also appears to be the only group to have retained *CamCYC3* genes (Figs. 5, 7). This means that this lineage maintains both an extra *CYC1* and an extra *CYC3* gene compared to most other Lobelioideae clades. The Impares corolla shape does differ from other groups in having large, broad ventral and lateral corolla lobes and greatly reduced, nearly scale-like dorsal lobes [70]. However, there are no data that tie this morphology with extra *CYC1* and *CYC3* gene copies to date.

The giant lobelias (U clade) primarily grow in tropical montane habitats around the globe and have synapomorphies of a tree-like habit, often with lignification, and are tetraploid with a chromosome number of *n* = 14 [7, 10, 71]. In the U clade, there are 3 subclades of *CamCYC1*, with the U1 clade grouping with other Neotropical, Australia, and South American Lobelioideae species sister to a clade including U2A and U2B. The current topology suggests separate duplications in Pacific Basin species (Fig. 3, green) and non-Pacific Basin species (Fig. 3, yellow); however, there was no bootstrap support for the relationships of these clades, so a single duplication could be shared across all the giant lobelias. These groups were difficult to tease apart because sequence divergence is minimal and they were amplified and cloned together, which resulted in some mixing of sequences among copies. Nevertheless, *CYC1* duplicates are maintained in the giant lobelias, and better sampling could shed light on the precise ancestor(s) of this clade. For instance, in the U1 clade, *Lobelia doniana* is sister to the rest, supporting the East Asian origin hypothesis of giant lobelias [46], although they are nested within a grade of North American species.

### Duplication of *CamCYC2* in Campanulaceae is highly associated with bilateral symmetry in Lobelioideae

*CamCYC2* genes are the orthologs of *CYCLOIDEA*, a gene which has been shown repeatedly to exhibit dorsally restricted expression in bilaterally symmetrical groups (see Hileman [39]). Additionally, the evolution of bilateral symmetry has been correlated with duplications in *CYC2* genes [39, 60]. These genes are of interest in bilaterally symmetrical species of Campanulaceae, where we expect gene expression to be restricted to one side of the flower and that duplications will likely be frequent. *CamCYC2* in Lobelioideae was well-sampled and, as expected, had a clear duplication across the entire clade (Figs. 4, 7). The *CamCYC2* duplication very likely occurred in the Lobelioideae ancestral lineage after it diverged from Campanulaceae sensu stricto. Both Lobelioideae *CamCYC2* gene clades share a similar pattern and are broadly congruent both with previous research [8, 10, 46, 47] and with the Lobelioideae *CamCYC1* gene clade. As in *CamCYC1*, we also detected duplications in the U subclade in both *CamCYC2* paralogs, likely due to tetraploidy. Flowers of Lobelioideae are resupinate, twisting their pedicel (Fig. 2A, C). However, since mature flowers, after turning, end up having a flower that looks right side up (i.e., a standard core eudicot 2 + 3 lobe arrangement); this suggests there is a developmentally earlier change in orientation to create an initial 3 up, 2 down lobe arrangement. Taxa such as species in *Monopsis* (G) do not twist their pedicel and end up with mature 3 + 2 flowers, reverting to the hypothesized ancestral Lobelioideae flower orientation. That said, *Monopsis* species did not lose their *CamCYC2* copies like Cyphioideae, which similarly does not undergo resupination. There are currently no known genes involved in twisting of plant tissues, for instance, to present the flower upside down, allowing us to potentially uncover novel gene functions of *CYC-like* genes with further studies of these groups.

Within both of the *CamCYC2A* and *CamCYC2B* clades, the U subclade (giant lobelias) occur in two duplicate locations in the phylogeny, likely due to their tetraploid ancestry [48, 49]. This means that there are four separate clades of *CYC2* in giant lobelias. One of the U clades in each of *CamCYC2A* and *CamCYC2B* have no clear sister group; however, the other clade in each is most-closely related to *Lobelia urens*. This relationship to *Lo. urens* is not well-supported in either clade, and *Lo. urens* is clearly part of a Mediterranean clade likely derived by amphitropical dispersal from what is now the Western Cape of South Africa [46]. Two species in the Tupa group of South America have evolved woody growth independently from the giant lobelias [5, 10, 46]. The hexaploid Tupa group [19] appears to have independently duplicated in *CamCYC2B*, similar to the giant lobelia (U) group.

### Gene expression of *CamCYC2* in Lobelioideae species is conserved following resupination and paralog dominance is correlated with dorsal petal size

**Fig. 8:**
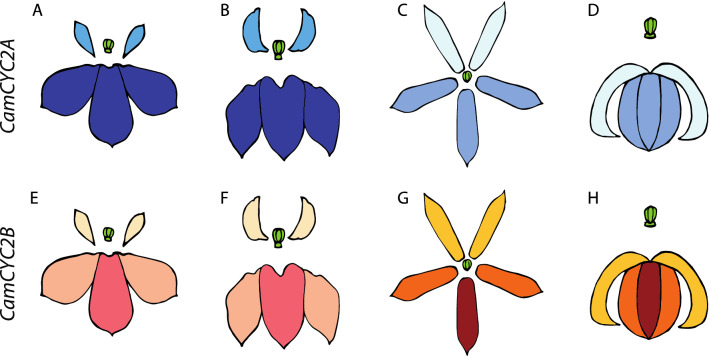
*CamCYC2A* and *CamCYC2B* expression pattern in Lobelioideae species. **A**–**D** *CamCYC2A* expression pattern, **E**–**F** *CamCYC2B* expression pattern. **A**, **E** *Lobelia erinus*, **B**, **F** *Lobelia siphilitica*, **C**, **G** *Lithotoma axillaris*, **D**, **H** *Lobelia polyphylla*. Low saturation of color represents minor expression, high saturation of color represents high expression in flower buds. *CamCYC2A* is more highly expressed in species with relatively small dorsal corolla lobes, (**A**, **B**) while *CamCYC2B* is the more highly expressed in species with relatively large dorsal corolla lobes (**G**, **H**). *CamCYC2A* is weakly expressed in the dorsal corolla lobes (the true ventral domain) and is highly expressed in the ventral domain (the true dorsal domain). *CamCYC2B* has weak expression in the dorsal corolla lobes (the true ventral corolla lobes), medium expression in lateral corolla lobes, and high expression in the ventral corolla lobe (the true dorsal corolla lobe)

In Lobelioideae species, we isolated two copies of *CamCYC2* genes and utilized qRT-PCR to examine their temporal and spatial expression patterns. As previous researchers have shown, *CYC2*-like genes are dorsally restricted, limited to the adaxial region of flower tissues. In most examined bilaterally symmetrical species, *CYC2*-like paralogs are diverged in their expression, with one copy being more restricted dorsally than the other [36, 39]. Lobelioideae have resupinate flowers and we hypothesized that *CamCYC2* expression would, like other bilaterally symmetrical flowers, be adaxial, corresponding to finally positioned ventral in these resupinate flowers. Using four Lobelioideae species, *Lobelia erinus*, *Lo. siphilitica*, *Lo. polyphylla*, and *Lithotoma axillaris*, we found that (1) the paralogs varied in how restricted they were on the dorsoventral axis, (2) that resupinate flowers led to the highest expression in finally positioned ventral regions, and (3) the overall patterns of expression among lobes was similar across species; however, which paralog exhibited greater expression varied (Figs. 6, 8).

The temporal expression patterns of *CamCYC2* genes were relatively uniform through development, which is similar to that observed in other groups [21]. The spatial expression patterns of *CamCYC2A* and *CamCYC2B* are relatively concordant among the four species (Figs. 6A-II, B-II, C-II, D-II, 8). *CamCYC2A* is expressed similarly in lateral and ventral lobes, or the whole ventral region of the flower (the adaxial region) and has very low expression in the dorsal lobes (abaxial initiation). *CamCYC2B* is always highly expressed in the ventral lobe (adaxial initiation), has an intermediate expression level in lateral lobes, and is barely expressed in dorsal lobes (abaxial initiation). Both *CamCYC2A* and *CamCYC2B* have little to no expression in the dorsal lobes, similar to leaf expression. A similar phenomenon is seen in Malpighiaceae, with a shift in the axis of the early flower primordia resulting in a rearrangement of floral petals in the New World Malpighiaceae species, which have 1 dorsal petal, 2 lateral petals, and 2 ventral petals. The expression *CYC2*-like genes in Malpighiaceae, however, remains in the dorsal regions of the corolla [56–58].

Previous work in Dipsacales has shown that even subtle differences in the dorsoventral gradient of *CYC2* expression is correlated with significantly different growth patterns of the lobes [40]. In *Lobelia erinus*, flowers with small dorsal lobes, *CamCYC2A* is significantly more expressed than *CamCYC2B* (Fig. 6A-II)*.* In *Lo. siphilitica*, flowers with relatively larger dorsal lobes than *Lo. erinus*, the pattern is the same, but the distinction between the level of expression is not as great, especially in the ventral lobe where the *CamCYC2B* gene expression level is almost 50% of the *CamCYC2A* gene expression level (Fig. 6B-II). In *Lithotoma axillaris* and *Lo. polyphylla*, there is no distinct difference in shape or size between dorsal, lateral, and ventral lobes. In an opposite pattern, the *CamCYC2B* gene is more highly expressed than the *CamCYC2A* gene (Fig. 6C-II, and D-II). This is effectively an increase in expression of the gene with the broader zone of expression, which has been shown to be correlated with a more radialized flower [36]. In flower primordia, *CYC* genes repress cell growth and control organ number, and in later stages, *A. majus CYC2* paralogs can also upregulate cell division [26, 28]. In this case, Lobelioideae species have relatively larger lateral and ventral lobes (the genetically adaxial region), likely due to the high *CamCYC2* gene expression. However, it does not easily explain how *Li. axillaris* and *Lo. polyphylla* flowers have lobes with almost the same size and shape. Nonetheless, this change in the expression ratio among paralogs sets up an intriguing system to study not just *CYC* function and evolution, but also how morphology can be substantially altered by shifts in expression dominance among gene paralogs.

## Conclusions

Campanulaceae are a large core eudicot clade that exhibits a variety of floral symmetries, including varying types of resupination and pollination syndromes. The family occurs nearly world-wide and has become a model for studying adaptive radiations in many locations. We sequenced all three core eudicot paralogs of *CamCYC* genes, *CamCYC1*, *CamCYC2*, and *CamCYC3* (Fig. 7). The *CamCYC1* genes duplicated in radially symmetrical Campanuloideae, but not in other bilaterally symmetrical flower subfamilies. As expected, *CamCYC2* genes duplicated in the Lobelioideae clade with bilaterally symmetrical flowers. However, we show for the first time a loss of *CYC2*-like genes in a bilaterally symmetrical group, with no sequences found in Cyphioideae. Instead of *CamCYC2*, we found a potential duplication of *CamCYC3* in this group. It is possible that, in Cyphioideae, *CamCYC*3 genes may have taken on the role of *CYC2*-like genes. Future studies examining floral RNA expression in *Cyphia* should be highly informative. Nonetheless, the genetic programming of floral symmetry appears to be independently derived in Cyphioideae and Lobelioideae, supporting the hypothesis that ancestral Campanulaceae were radially symmetrical.

In Lobelioideae, expression patterns of *CamCYC2* genes were similar to previous studies across core eudicots species, with *CamCYC2A* and *CamCYC2B* both highly expressed in the adaxial side of flower related to meristem orientation (Fig. 8), despite resupination resulting in a ventral presentation in the flower, suggesting conservation of dorsal identity in these upside-down flowers. In addition, the *CamCYC2A* and *CamCYC2B* show distinctly different expression patterns in species with a different dorsal lobe size ratio. *CamCYC2A* is the dominant *CamCYC2* gene in species with smaller dorsal lobes, like *Lobelia erinus*, and *Lo. siphilitica*. *CamCYC2B* is the dominant *CamCYC2* gene in species with bigger dorsal lobes, in which the dorsal lobes are almost the same size as the lateral and ventral lobes, like *Lithotoma axillaris* and *Lo. polyphylla*. We illustrate here for the first time that *CYC* expression is conserved along the dorsoventral axis of the flower even as it turns upside-down, suggesting that at least later *CYC* expression is not regulated by extrinsic factors such as gravity. Additionally, the shift in expression dominance among paralogs provides intriguing data that differences in ratios of expression in *CYC* could lead to shifts in morphological growth ratios in the flower.

## Materials and methods

### Sampling and plant materials

We examined a total of 132 DNA samples from 128 species, including nine Cyphioideae species, nine Campanuloideae species, and 110 Lobelioideae species. Table 1 provides DNA source information for previously prepared samples. An additional eight Campanuloideae and eight Lobelioideae species were from live plants growing in the greenhouse at St. John’s University. *Campanula persicifolia* was wild collected in Fresh Meadow, New York. *Campanula carpatica* was bought from a local nursery garden. *Campanula glomerata*, *Campanula portenschlagiana*, *Campanula cochleariifolia*, *Jasione montana*, *Platycodon grandiflorus*, *Phyteuma scheuchzeri*, *Lobelia anceps*, *Lobelia bridgesii*, *Lobelia cardinalis*, *Lobelia erinus*, *Lobelia siphilitica, Lobelia polyphylla*, *Lobelia tupa*, and *Lithotoma axillaris* seeds were ordered from online plant nurseries (Botanical Interests ®, Hazzard’s Seeds, and Plant World Seeds). All DNA was extracted using a DNeasy Plant Mini Kit (QIAGEN) according to the manufacturer’s instructions and stored in – 20 °C.

### Amplification

All PCR reactions were performed using *Taq* DNA Polymerase (Go*Taq*® Flexi DNA polymerase, Promega). All DNAs were amplified in 25 μL PCR reactions containing: 1 μL DNA, 5 μL 5× buffer, 2.5 μL 25 mM MgCl_2_, 0.5 μL 10 mM dNTPs, 1 μL of 10 mM primers, 1 μL *Taq* polymerase, and distilled water was added to bring up to total volume. Amplifications utilized the following cycling program: (1) initial denaturation was carried out at 94 °C for 2 min; (2) 39 cycles of: 94ºC for 45 s, 51 °C (varied for by different pairs of primers) for 1 min, and 72 °C for 1 min 30 s; (3) a final elongation step at 72 °C for 20 min. To amplify *CYC*-like genes in Campanulaceae, previously designed degenerate primers were used from Howarth and Donoghue [41]. Primers for *CamCYC1* were designed based on *CYC*-like sequences from other lineages in asterids available from NCBI. All primer sequences are provided in Table 2.

**Table 2:** Primers for different *CYC* paralogs in Campanulaceae

Locus	Primer	Primer sequences (5′-3′)
*CYCLOIDEA1*	Astl CYC1Fa	CGRAGRATGAGRYTRTCNCTTGATG
	Astl CYC1Ra	GCCCTTKCYCTTGCYCTTTCCCTTG
*CYCLOIDEA2*	CYC73b	GCNCGNARRTTYTTYGATCTDCAAG
	CYCRa	CTTGCTCTTTCYCTYGCYTTYGCCC
*CYCLOIDEA3*	CYC73b	GCNCGNARRTTYTTYGATCTDCAAG
	CYCRa	CTTGCTCTTTCYCTYGCYTTYGCCC
	Astl CYC3Fa	GGGAAGAMAGAYMGGCAYAGC
	Astl CYC1Ra	GCCCTTKCYCTTGCYCTTTCCCTTG

Cloning was performed using the StrataClone PCR Cloning Kit (Agilent, Santa Clara, CA), following the manufacturer’s instructions. We picked four to eight colonies per plate and amplified them using primer sites in the construct (M13F and M13R). DNA cleaning utilized the P.E.G. method [72]. Sanger sequencing was performed at the Yale University DNA Analysis Facility, New Haven, CT, using a 3730xl DNA Analyzer (Applied Biosystems, Thermo Fisher Scientific, Inc.).

### Alignment and phylogenetic analyses

All individual colony sequences were edited in Geneious® Pro v.7.1.2 (http://www.geneious.com), including removing the plasmid and primer sequences. Consensus sequences were generated from similar clones from the same DNA sample. To determine orthology, we initially used BLAST in NCBI. *CYC*-like genes were determined by the presence of the TCP and R domains. The species-level consensus sequences were aligned in Geneious® using the MUSCLE Alignment tool (default parameters) and then manually adjusted according the amino acid sequences or nucleotide sequences in Mesquite [73] or Geneious® Pro v.7.1.2. The phylogenetic gene trees were generated with CIPRES science gateway (https://www.phylo.org) using Maximum Likelihood by the RAxML-HPC BlackBox (default parameters, except with added option to let RA×ML halt bootstrapping automatically and estimate the proportion of invariable sites (GTRGAMMA + I)). Phylogenetic trees of *CYC1* and *CYC3* were midpoint rooted, while in *CYC2* the Campanuloideae sequences were used as an outgroup to Lobelioideae sequences.

### Collection and dissection of floral tissues

For expression studies, four Lobelioideae species were grown in the greenhouse at St. John's University, Queens, NY, USA: *Lobelia erinus* (Fig. 1H), *Lo. siphilitica* (Fig. 1G), *Lo. polyphylla* (Fig. 1I), and *Lithotoma axillaris* (Fig. 1F). Living collections are maintained at SJU greenhouse and herbarium specimens are deposited at NYBG. Flower buds were collected at three different developmental stages: small buds, medium buds, and large buds. The small buds of *Lo. erinus* were 2.5–4 mm, medium buds were 5–6 mm, and large buds were 7–8 mm. For *Lo. siphilitica*, small buds were 5–6 mm, medium buds were 8–12 mm, and the large buds were 14–18 mm. For *Lo. polyphylla*, small buds were 7–10 mm, medium buds were 15–20 mm, and the large buds were 25–30 mm. For *Li. axillaris*, small buds were 10–13 mm, medium buds were 15–25 mm, and the large buds were 25–35 mm. Additionally, medium flower buds were dissected, after resupination, to separate the finally positioned dorsal, lateral, and ventral corolla lobes. Leaf tissue was separately collected as a control. All tissues were immediately frozen with liquid nitrogen and stored in a – 80 °C freezer until extraction. Roughly 20–30 mg of tissue was collected for each RNA extraction. The exception was tissue from *Lo. erinus* flower buds, which are extremely small, with 3–4 mm medium size buds, so therefore, only roughly 15–20 mg was collected for RNA extraction in this species. Three biological replicates were collected for each type of tissue.

### Quantitative Real-Time PCR and statistical analysis

Total RNA was extracted from plant tissues used for qRT-PCR using the RNeasy Plant Mini Kit and RNase-free DNase kit (QIAGEN) according to the manufacturer’s instructions and then stored at -80 °C. The concentrations and purities of all RNA samples were determined using a Thermo Scientific NanoDrop 2000 (Thermo Scientific, Waltham, MA). The qRT-PCR primers were designed in Geneious® Pro v.7.1.2 based on *CamCYC2* gene sequences and ACTIN sequences collected in our study. Specific primer sets were designed for each species (Table 3). The qScript™ One-Step SYBR® Green qRT-PCR Kit (QuantaBio) was used with manufacturer recommendations to investigate the expression patterns of *CamCYC2A* and *CamCYC2B* gene expression in the collected tissues from *Lobelia erinus*, *Lo. siphilitica, Lithotoma axillaris,* and *Lo. polyphylla*. Each type of tissue included three biological and two technical replicates. Samples were run on a Bio-Rad MyIQ Single Color Real-Time RCR Detection System (Bio-Rad, Hercules, CA). The melting curve and threshold cycle (Ct) values were analyzed by a modified 2^−ΔC^_T_ method [74]. Because all of the tissues used were from natural or wild-type plants, there was no “untreated control” to normalize the second delta as is standard in these methods. ANOVA and post hoc Tukey HSD were performed on the web site: https://astatsa.com/OneWay_Anova_with_TukeyHSD/.

**Table 3:** The *q*RT-PCR primers for each species examining *CamCYC2A* and *CamCYC2B* gene expression patterns

		Efficiency (%)
	*Lobelia erinus*	
*CamCYC2A*	CYC2 37F 5′-GCTAGTAAAACCCTTGATTGGCT-3′	82.7
	CYC2A 314R 5′-GCCCTGGACTCTTTTGCAAAGT-3′	
*CamCYC2B*	CYC2 37F 5′-GCTAGTAAAACCCTTGATTGGCT-3′	81.8
	CYC2B 291R 5′-GCGATGAGATGCAGGTTTATAACTG-3′	
*CamActin*	Le-act1046F 5′-ATCCACGARACSACCTACAACT-3′	86.0
	Le-act1216R 5′- MACCACCTTAATCTTCATGCTGCT-3′	
	*Lobelia siphilitica*	
*CamCYC2A*	ls CYC2A-37F 5′-TTCGACAAAGCTAGTAAAACTCTTGATTGG-3′	81.6
	Ls CYC2A 264R 5′-TTTCTCTTTGGCTCTCGTTGTAGC-3′	
*CamCYC2B*	ls CYC2B-47F 5′-CTAGTAAAACCCTTGATTGGCTTTTCAC-3′	87.0
	Ls CYC2B 298R 5′-CTAGGCGATGAGATGCAGGTTTATAAC-3′	
*CamActin*	Le-act477F 5′-AGATYTGGCATCAYACTTTCTACA-3′	89.0
	Le-act729R 5′- CCTTCGTARATTGGAACCGTGTG-3′	
	*Lithotoma axillaris*	
*CamCYC2A*	Ls CYC2A F41 5′-ACAAAGCTAGTAAAACTCTTGATTGGCT-3′	89.3
	ISCYC2A 260R 5′-TTCTCTTTGGCGCTCGATGTAGCTG-3′	
*CamCYC2B*	ISCYC2B 38F 5′-TTGACAAAGCTAGTAAAACCCTTGATTGG-3′	92.8
	ISCYC2B 203R 5′-GCTCCTTCATTTTGTTCAGCTGC-3′	
*CamActin*	Le-act1046F 5′-ATCCACGARACSACCTACAACT-3′	86.0
	Le-act1216R 5′-MACCACCTTAATCTTCATGCTGCT-3′	
	*Lobelia polyphylla*	
*CamCYC2A*	LP CYC2AF1a 5′-TCGACAAAGCTAGTAAAACTCTTGATTGG-3′	89.6
	LP CYC2A R4 5′-TTTGCAAGATAAAGTGCAGGTTTATACG-3′	
*CamCYC2B*	Ls CYC2B F43 5′-AAAGCTAGTAAAACCCTTGATTGGCT-3′	85.7
	LP CYC2B R1 5′-TTTGTGCTCTCATCGTTTTCGCTTCAC-3′	
*CamActin*	Le-act477F 5′-AGATYTGGCATCAYACTTTCTACA-3′	89.0
	Le-act729R 5′-CCTTCGTARATTGGAACCGTGTG-3′	

The annealing temperature for all primers is 60 ℃

## Data Availability

The datasets supporting the conclusions of this article are available in the TreeBase repository (https://www.treebase.org/treebase-web/login.jsp) and GenBank repository (XX-XX); not public until accepted).

## Abbreviations

CYC: CYCLOIDEA
TCP: Teosinte Branched1, CYCLOIDEA, proliferating cell factor
qPCR: Quantitative real-time polymerase chain reaction
RT-PCR: Reverse transcription polymerase chain reaction
ANOVA: Analysis of variance
HSD: Honest significant difference
Ct: Threshold cycle
CBS: Group of Lobelioideae including *Centropogon*, *Burmeistera*, and *Siphocampylus*
